# Selection and characterization of alanine racemase inhibitors against *Aeromonas hydrophila*

**DOI:** 10.1186/s12866-017-1010-x

**Published:** 2017-05-25

**Authors:** Yaping Wang, Chao Yang, Wen Xue, Ting Zhang, Xipei Liu, Jiansong Ju, Baohua Zhao, Dong Liu

**Affiliations:** 1 College of Life Science, Hebei Normal University, Shijiazhuang, China; 2 Department of Chemistry, New York University, New York, NY 10003 USA

**Keywords:** *Aeromonas hydrophila*, Alanine racemase, Inhibitor, Molecular docking

## Abstract

**Background:**

Combining experimental and computational screening methods has been of keen interest in drug discovery. In the present study, we developed an efficient screening method that has been used to screen 2100 small-molecule compounds for alanine racemase Alr-2 inhibitors.

**Results:**

We identified ten novel non-substrate Alr-2 inhibitors, of which patulin, homogentisic acid, and hydroquinone were active against *Aeromonas hydrophila*. The compounds were found to be capable of inhibiting Alr-2 to different extents with 50% inhibitory concentrations (IC_50_) ranging from 6.6 to 17.7 μM. These compounds inhibited the growth of *A. hydrophila* with minimal inhibitory concentrations (MICs) ranging from 20 to 120 μg/ml. These compounds have no activity on horseradish peroxidase and d-amino acid oxidase at a concentration of 50 μM. The MTT assay revealed that homogentisic acid and hydroquinone have minimal cytotoxicity against mammalian cells. The kinetic studies indicated a competitive inhibition of homogentisic acid against Alr-2 with an inhibition constant (*K*_i_) of 51.7 μM, while hydroquinone was a noncompetitive inhibitor with a *K*_i_ of 212 μM. Molecular docking studies suggested that homogentisic acid binds to the active site of racemase, while hydroquinone lies near the active center of alanine racemase.

**Conclusions:**

Our findings suggested that combining experimental and computational methods could be used for an efficient, large-scale screening of alanine racemase inhibitors against *A. hydrophila* that could be applied in the development of new antibiotics against *A. hydrophila*.

**Electronic supplementary material:**

The online version of this article (doi:10.1186/s12866-017-1010-x) contains supplementary material, which is available to authorized users.

## Background

*Aeromonas hydrophila* is a gram-negative facultative anaerobic bacterium of major public health concern that causes a variety of diseases in both fish and humans, resulting in severe economic losses [1]. Extensive antibiotic use has led to antibiotic resistance, which can potentially be transferred to other aquatic bacteria and human pathogenic bacterial strains [2].

Thus, there is considerable interest in the identification and development of targets for drug design. One such target is alanine racemase [3, 4]. Alanine racemase (EC 5.1.1.1) is a pyridoxal-5′-phosphate (PLP)-containing homodimeric enzyme that catalyzes the interconversion of L-alanine to D-alanine [5]. D-Alanine is an essential building block of the cell wall of both gram-positive and gram-negative bacteria. There are no known homologs of alanine racemases in humans, but because they are ubiquitous among prokaryotes, they make an attractive antimicrobial target [6, 7].

Numerous inhibitors, such as *O*-carbamyl-d-serine, d-cycloserine, β,β-trifluoroalanine, alanine phosphonate, L-amino-cyclopropane phosphonate, and β-chloro- and β-fluoro alanine, were identified as able to affect the activity of alanine racemase [8, 9]. All of these inhibitors were structural analogs of alanine: they interact with the enzyme-bound PLP and interfere with the catalytic process of the enzyme, but they lack target specificity with a tendency to inactivate other unrelated PLP-dependent enzymes, leading to cellular toxicity [10]. PLP-related off-target effects could be overcome by using enzyme inhibitors that are not substrate analogs.

The PLP cofactor enables the enzyme to lower the pKa of the α-proton by stabilizing the attendant carbanion using a substrate-bound external aldimine [11]. The majority of substrate analogs, such as D-cyloserine, engage PLP and lack target specificity, so the strategies for screening alanine racemase inhibitors with an improved impact include targeting the PLP-independent inhibitors that bind to the dimer interface and block dimerization or by placing an inhibitor at the entrance of the catalytic pocket, thereby blocking substrate entry. A promising strategy may be to design or screen inhibitors that bind to the catalytic pocket, targeting the active sites [3].

To discover promising alanine racemase inhibitors that will be useful for developing a novel antibiotic, we proposed a combination experimental and computational screening method. First, we established a screening assay for the identification of small-molecule inhibitors in a 96-well format using a library of 2100 compounds, followed by antimicrobial susceptibility against *A. hydrophila*, cellular cytotoxicity activity and kinetic studies on the inhibitors. Additionally, the mode of interaction between the inhibitors and the Alr-2 protein was modeled using molecular docking techniques. With these techniques, we developed a novel alanine racemase inhibitor screening method.

## Methods

### Bacterial strains, plasmid and culture conditions

*A. hydrophila* HBNUAh01 isolated from infected *Paralichthys olivaceus* [12] and an *A. hydrophila alr*-2 knockout mutant [13] were used in this study. *Escherichia coli* BL-21(DE3) cells were used for protein expression. The pET-25b-*alr*2 plasmid [14] was used for protein expression. The *A. hydrophila* and *E. coli* strains were cultured in Luria-Bertani (LB) medium at 30 °C and 37 °C, respectively. For plasmid selection, 0.5 mmol/l ampicillin (AMP, Sigma–Aldrich Inc., USA) was added to the LB medium for experiments with *E. coli*.

### Compounds and cell culture

Patulin, d-cycloserine, homogentisic acid and hydroquinone were all purchased from Sigma (Sigma–Aldrich Inc., USA). The HeLa cell line (CCL2 from ATCC) was cultured in RPIM1640 supplemented with 10% fetal calf serum and a mixture of antibiotics (Penicillin 10^5^ U/L, Streptomycin 100 mg/L) under a 5% CO_2_ atmosphere.

### Purification of alanine racemase

Alanine racemase was produced and purified as described previously [14]. Pre-cultured BL-21 (DE3) cells with the *alr*-2 gene cloned in pET-25b (+) (2 ml) were inoculated into 100 ml of fresh LB culture at 37 °C. Protein overproduction was induced when the cell density at OD_600_ reached 0.6 by the addition of IPTG at a final concentration of 1 mM. The induced cultures were incubated overnight at 28 °C.

The cells were harvested, resuspended into 20 ml of sample buffer (50 mM NaH_2_PO_4_, pH 8.0, 300 mM NaCl, and 10 mM imidazole), and lysed by sonication until a clear lysate was obtained. The crude lysate was centrifuged, and the cell-free supernatant was mixed with 2 ml of 50% Ni-NTA agarose slurry (Qiagen, Germany) and incubated at 4 °C for 1 h. Unbound proteins were washed three times with 4 ml of buffer. Bound proteins were eluted four times with 0.5 ml of buffer.

The purified protein was concentrated and dialyzed against the same buffer with 10% glycerol by ultrafiltration with an Amicon Ultra-15 Centrifugal Filter Device (30 K MWCO, Millipore). The purity and molecular weight of the enzyme were determined by sodium dodecyl sulfate polyacrylamide gel electrophoresis (SDS–PAGE).

### Adaptation of the alanine racemase enzyme assay to a 96-well format

**Fig. 1:**
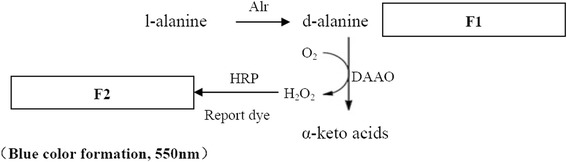
Assays for screening alanine racemase-specific inhibitors. Alanine racemase converts l-alanine to d-alanine, providing a substrate for d-amino acid oxidase, which produces hydrogen peroxide. The combination of hydrogen peroxide, horseradish peroxidase, and a dye molecule leads to an insoluble colored product. Fluorescence intensity was measured in a microplate reader

Alanine racemase activity was measured at room temperature in the L → D direction using a spectrophotometric assay [14]. The assay was modified by varying the volume to 100 μl and dividing it into two steps (Fig. 1).

The first step (F_1_) is the production of the d-form amino acids. The optimized enzyme assay reaction mixture consisted of 10 μM PLP, 12.5 mM of NaHCO_3_-NaOH buffer (pH 11), and 50 mM l-alanine and alanine racemase. A total of 100 μl of this reaction mixture was added to the wells of a 96-well plate. After incubation at 35 °C for 10 min, the reaction was terminated by adding 12.5 μl of 2 M HCl, and the incubation was continued for 2 min on ice. The reaction mixture was centrifuged at 10,000 RPM, for 10 min at 4 °C, and 10 μl of 2 M NaOH was added to neutralize the reaction mixture. The second step (F_2_) is the conversion of d-alanine to α-keto acids using d-amino acid oxidase (DAAO). The formation of H_2_O_2_ from this reaction can be quantified using horseradish peroxidase (HRP) at 550 nm. The DAAO reaction mixture contained 200 mM of Tris-HCl (pH 8.0), 0.1 mg/ml 4-aminoantipyrine (AAP), 0.1 mg/ml *N*-ethyl-*N*-(2-hydroxy-3-sulfopropyl)-*m*-toluidine sodium salt (TOOS), 2 units of HRP, 0.1 unit of DAAO and d-form amino acid solution in a final volume of 100 μl. The negative control for the enzyme assay was prepared using the same procedure but without enzyme. After incubating at 37 °C for 30 min, the absorbance was measured at 550 nm using a Spectra MAX 190 microplate reader (Molecular Devices Corp., USA). One unit (U) of enzyme was defined as the amount of purified enzyme that catalyzed the formation of 1 μmol of d-alanine from l-alanine per minute.

### Inhibitor screening procedure

Each well (sample, control, and their blanks) contained the screening assay reaction mixture of 10 μM PLP and 12.5 mM NaHCO_3_-NaOH buffer (pH 11). Next, 4 μl of test substance (small molecular compounds or natural product extracts) was added to the sample and sample blank test tubes. Finally, 2 mM alanine racemase was added to the sample and control test tubes while the same volume of DMSO was added to the sample blank and control blank test tubes. After incubation at 4 °C for 30 min, 50 mM l-alanine was added to the sample and control test wells while DMSO was added to the sample blank and control blank test wells to obtain a final volume of 100 μl. The control and control blank were defined as 100% and 0% of enzyme activity. The reaction was then carried out as described for the alanine racemase assay. The assay is divided into two steps: absorbance was measured at 550 nm at the end of the first (F_1_), and second (F_2_) steps and ΔF was defined as F_2_-F_1_. Inhibition activity was calculated from the following equation.

$$ \mathrm{Inhibition}\kern0.5em \left( I\%\right)\kern0.5em =\kern0.5em \frac{\left[\Delta \mathrm{F}\kern0.5em \left(\mathrm{control}\right)\kern0.5em \hbox{--} \kern0.5em \Delta \mathrm{F}\kern0.5em \left(\mathrm{control}\ \mathrm{blank}\right)\right]\kern0.5em -\kern0.5em \left[\Delta \mathrm{F}\ \left(\mathrm{sample}\right)\kern0.5em \hbox{--} \kern0.5em \Delta \mathrm{F}\ \left(\mathrm{sample}\ \mathrm{blank}\right)\right]}{\Delta \mathrm{F}\kern0.5em \left(\mathrm{control}\right)\kern0.5em \hbox{--} \kern0.5em \Delta \mathrm{F}\kern0.5em \left(\mathrm{control}\ \mathrm{blank}\right)}\kern0.5em \ast 100\% $$

Two parameters were used to indicate the quality of the assay: signal-to-noise ratio (S/N) and the signal-to-background ratio (S/B). The S/N ratio is classically defined as S/N = (mean signal – mean background)/standard deviation of background. The S/B ratio is classically defined as S/B = mean signal/mean background. The Z′ factor is a well-established measure of the assay’s quality or suitability, as described previously [15]. The Z′ factor is defined by the following formula: Z′ = 1 – [3(SD sample + SD control)/ (Mean sample – Mean control)], where SD is the standard deviation, the control is the control blank, and M is the mean.

### Compound library

A library of 2100 natural compounds and the fungal fermentation broths were stored in solution with DMSO. The compound library is from the new drug research and development Co., Ltd. of the North China Pharmaceutical Corporation. The library consists of compounds that were mainly from metabolites of microorganisms.

### Enzyme IC_50_ determination

Fourfold dilution series (in DMSO) were prepared for all the compounds, and the solutions were added to the wells of a 96-well plate to yield the final inhibitory concentrations. Each concentration was tested in triplicate. The substrate was added after incubation for 30 min, and the fluorescence intensity was measured after the reaction. The positive control (d-cycloserine, DCS) was diluted in DMSO, and the negative control was prepared without adding an inhibitor to the control wells of each plate. Percentage inhibition at each inhibitor concentration was calculated with respect to the negative control. The results were analyzed using the SPSS 16.0 IBM modeler to calculate the compound concentration that causes 50% inhibition (IC_50_).

### Assay interference

To eliminate the inhibitory effects of the compounds on DAAO and HRP, a counter screening assay was performed as described above. The first-step assay mixtures without alanine racemase and l-alanine were added to each test well, followed by the addition of a twofold dilution series (in DMSO) of the inhibitors to the sample test wells, and finally, the addition of DMSO to the blank control wells. Then, DMSO was added to each well to obtain reaction mixtures at a final volume of 100 μl.

Second, the d-amino acid oxidase reaction mixture was prepared using 10 μM D-ala as the substrate. The control and control blank for the enzyme assay was prepared with or without 10 μM D-ala and absorbance was measured at 550 nm.

### Antimicrobial susceptibility tests against *A. hydrophila*

*A. hydrophila* was cultured for 18 h, washed with PBS (pH 7.2), and adjusted to an OD_600_ value of 0.5. Next, the culture was diluted tenfold five times, and aliquots were spread on LB agar in triplicate to determine the number of colony-forming units (CFU)/ml.

The minimum inhibitory concentration (MIC) of the chemical compounds against *A. hydrophila* was determined using the microdilution method in accordance with the guidelines of the Clinical and Laboratory Standards Institute, document M31-A3 [16], following the method described by Dal Pozzo et al. [17]. Compounds were diluted in DMSO at concentrations of 80, 40, 20, or 10 μg/ml. Appropriate controls were included in all tests. DCS is a naturally occurring antibacterial compound that targets alanine racemase involved in peptidoglycan synthesis [18]. DCS was used as a positive control (50 and 100 mg/ml), DMSO solvent was used as a negative control for growth inhibition and DMSO alone was used as the blank control. All tests were performed in triplicate. The inoculum was prepared in LB culture medium (1 × 10^8^ CFU/ml; OD_600_ = 0.3) and cultured at 30 °C/20 h. The inoculum (100 μl; 1 × 10^5^ CFU) was added to each well containing compounds. The microplates were incubated at 30 °C for 20 h.

### Compound cytotoxicity studies

This assay was performed in a 96-well plate format and used HeLa cells [19]. The cell viability was determined using 3-(4,5-dimethyl-2-thiazole)-2,5-diphenyl-2H-tetrazolium bromide (MTT, Sigma-Aldrich). Cells were seeded in culture medium in microplates (4000 cells/well) and incubated at 37 °C for 24 h before drug treatments. Compounds were diluted in culture medium to final concentrations of 200, 100, 50, 25, 12.5, or 6.25 μg/ml and added to the cells. The cells were exposed to the compounds for 48 h. At the end of the incubation, the cells were exposed to MTT (0.5 mg/ml) at 37 °C for 4 h. The reduced crystals were dissolved in DMSO, and absorbance was detected at 490 nm. The control wells were set as zero absorbance. The percentage of cell survival was calculated using the background-corrected absorbance as follows: Cell survival (%) = (OD_experiment_/OD_control_) × 100. The data represent the mean and standard deviation from triplicate determination. The TC_50_ (the compound concentration that causes 50% cell death) was calculated using SPSS 16.0 software.

### Kinetics of alanine racemase inhibition

The mode of inhibition of the enzyme by the compounds was determined as follows. The experiment was composed of three sets of reactions in which each set consisted of four concentrations of substrate in the presence of fixed amounts of alanine racemase, and three different concentrations of inhibitors were used. For homogentisic acid and hydroquinone, 0, 0.02 and 0.04 mg/ml were used. The reactions were made as described [14]. The amount of product was determined spectrophotometrically and subsequently the standard curve was used to obtain reaction velocities. A double reciprocal plot (1/V versus 1/[S]), where V is reaction velocity and [S] is substrate concentration, was plotted. The type (mode) of inhibition of enzyme activity by the compounds was determined by analysis of the double reciprocal (Lineweaver-Burk) plot using Michaelis-Menten kinetics. The inhibition constant (*K*_i_) of the inhibitors was determined.

### Molecular docking

The flexible molecular docking method AutoDock [20] was used to analyze the intermolecular interaction between the Alr-2 protein and the small-molecule inhibitors. In the protocol, the alanine racemase Alr-2 structure was constructed with homology modeling (http://www.swissmodel). The crystal structure of AlaR (PDB IDs: 2rjh.1.A) [21], which has 55.4% amino acid similarity with Alr-2, was used as a template. The models were built using SWISS-MODEL in the automatic modeling mode and with default parameters. The quality of the models was evaluated using QMEAN and GMQE [22]. To prepare for docking, Gasteiger and Kollman united atom charges were assigned for the inhibitor molecule and the Alr-2 protein, respectively. A polar hydrogen atom was also added to the protein. A grid was set to accommodate the active site region with a 0.375 Å span. The torsion and rotatable bonds in the ligands were defined, and the nonpolar hydrogens and partial atomic charges were added to the bonded carbon atoms [23]. The docking was carried out using the AutoDock Vina program [24] to evaluate ligand binding energies over the conformational search space using the Lamarckian genetic algorithm.

### Statistical analysis

All experimental designs, statistical analyses and graphical representations of data were generated using SPSS 16.0 (SPSS Inc., USA) and the Prism 6.0 software program (GraphPad Software Inc., USA). IC_50_ data were analyzed by non-linear regression analysis. The *P* values in the experiments were obtained by using a 2-tailed *t* -test. A *P* value of <0.05 was considered statistically significant.

## Results

### Inhibitor screening results

The assay was adapted from a 200 μl format to a 100 μl 96-well plate format and optimized. Normal activity was determined in the reaction system and performed in triplicate, with an average OD value of 0.689. Then, the reaction system was divided in half while maintaining the concentration of each component, and the enzyme activity was determined, with the average OD value of 0.68. The S/B and S/N ratios of the assay system were found to be 28 and 32.7, respectively; the calculated value for the screening window coefficient (Z′ factor) was 0.83. The data indicated that the assay was suitable for inhibitor screening [15].

**Fig. 2:**
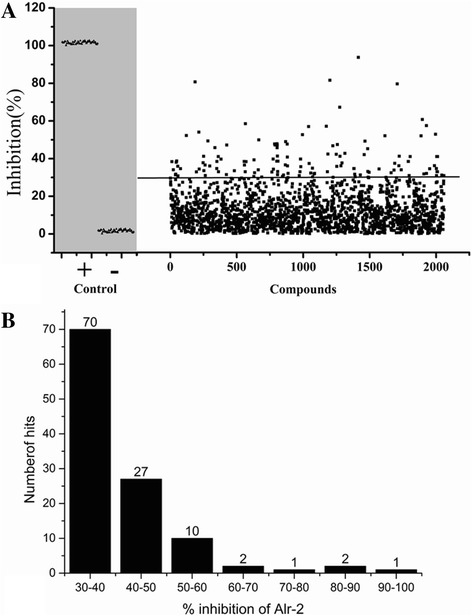
Summary of the screening results. **a** Results of positive and negative controls for 96 representative wells of the alanine racemase assay. The positive control represents 100% inhibition (wells 1–48), and the negative control represents 0% inhibition in the presence of 1% DMSO (wells 49–96). Data shown are of two replicates for the entire 2100 compound screen obtained from 96-well plates. **b** Distribution of the 113 hits, with the hits categorized into 7 groups with respect to percent inhibition

**Fig. 3:**
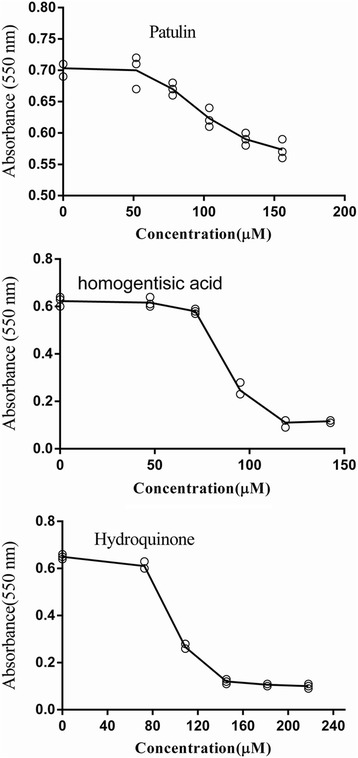
Counter-screen of three inhibitors. Concentration–response plots are shown for three enzyme inhibitors, (**a**) patulin; (**b**) homogentisic acid; (**c**) hydroquinone. No assay interference was noted for these reagents within a concentration of 50 μM

The library of 2100 compounds was screened in duplicate using final concentrations of 20 and 50 μg/μl for compounds and fungal fermentation products, respectively. A graphical representation of the screening results is shown in Fig. 3. Based on a report by Anthony KG et al. [6], compounds showing greater than a 30% inhibition with respect to the control and control blanks (Fig. 2a) were considered preliminary “hits”. We identified 111 compounds from the synthetic compound libraries and 2 compounds from the fungal fermentation broths as hits (Fig. 2a). Based on the percent inhibition of alanine racemase, there were 3 strong (>80%), 13 intermediate (51–80%), and 97 weak (30–50%) inhibitors detected in the library (Fig. 2b).

### Further selection criteria for hits and determination of the IC_50_ of alanine racemase

All of the hits were retested in a fourfold dilution series. Ten of these compounds specifically inhibited alanine racemase in a dose-dependent manner with more than 50% inhibition at the highest dose. Table 1 shows the chemical structures of the 10 new alanine racemase inhibitors and their IC_50_ values. The screening data of ten compounds are shown in the Additional file 1: Figure S1. The results showed that the IC_50_ values ranged from 6.6 to 18.5 μM, with the most potent activity shown by compound I-1. Although these compounds showed high inhibition against alanine racemase, the IC_50_ values of the compounds were 1.2 to 3.4 times higher than that of DCS.

**Table 1:** The inhibition values of active compounds for Alr-2

Hits	Inhibitor	MW		Structure	^a^IC50 (μM)	^b^IC50 (μM)	^c^MIC (μg/ml)	^d^MIC (μg/ml)	^e^TC_50_ (μg/ml)	^f^T_i_
I-1	Anabellamide	_583.626_			6.6(0.19)		NC	NC		
I-2	Patulin	154.12			14.7(0.27)		20(0.89)	80(3.96)	NC	NC
I-3	Homogentisic acid	168.147			12.5(0.2)		120(1.73)	NC	157.7(6.1)	1.3
I-4	Acetic acid,[4-(5-butyl-5-methyl-2(5H)-furanylidene)dihydro −3,5-dioxo-2(3H)-furanylidene]-(9CI)	292.284			8.9(0.34)		NC	NC		
I-5	Propyl Gallate	212.2			8.6(0.5)		NC	NC		
I-6	Hydroquinone	110.11			18.5(0.18)		80(2.6)	140(2.4)	122.9(8.9)	1.54
I-7	Benzenepropanoic acid,2-(9-acridinylamino)- monohydrochloride	378.85			17.7(0.34)		NC	NC		
I-8	Haematoxylin	302.29			15.6(0.23)		NC	NC		
I-9	Higenamine	271.32			14.3(0.21)		NC	NC		
I-10	Quercetin	302.24			15.5(0.33)		NC	NC		
	DCS	102.09			5.4(0.3)		25(1.98)	50(2.9)	149.6(5.9)	5

NC: non calculable as the values are too high

^a^IC50 against Alr-2 with PLP, average values with standard deviations

^b^IC50 against Alr-2 without PLP, average values with standard deviations

^c^MIC against *A.hydrophila*, average values with standard deviations

^d^MIC against *A.hydrophila Δalr*-2, average values with standard deviations

^e^Cytotoxicity in HeLa cells, average values with standard deviations

^f^Ti = TC_50_/^1^MIC

### Antimicrobial activity of alanine racemase inhibitors against *A. hydrophila*

The ten compounds from above were screened for antimicrobial activity against *A. hydrophila*. The average values with standard deviations in parentheses are shown in Table 1. Three compounds (I-2, I-3, and I-6) were found to inhibit the growth of *A. hydrophila* significantly. To corroborate these findings, we proceeded to determine the MIC of these compounds using the micro-broth dilution method. Their MICs ranged from 40 to 120 μg/ml. Compound I-2 and DCS exhibited a similar MIC.

In our previous paper with *A. hydrophila Δalr*-2, knockout of the *alr*-2 gene resulted in cell wall damage and enhanced membrane permeability under D-alanine starvation [12]. These results indicated that the alanine racemase Alr-2 is important for *A. hydrophila*. To determine if the observed growth inhibition was due to the inhibition of alanine racemase Alr-2, we measured the antimicrobial activity against *A. hydrophila Δalr*-2*.* We found that there was a 2-fold increase in the MIC observed for DCS; further, we found a 4-fold and 0.75-fold increase for I-2 and I-6, respectively. I-3 had no antimicrobial activity against *A. hydrophila Δalr*-2*.* These results suggest that the antimicrobial activities of I-2 and I-6 might not be due solely to the inhibition of Alr-2 and that the antimicrobial activity of I-3 was only due to the inhibition of Alr-2 (Table 1).

### Assay validation

To illustrate the counter screen in the second step of the assay, the concentration–inhibition plots for three compounds are indicated (Fig. 3). Compound I-2 has a weak inhibition of the enzymes in the second step; the IC_50_ values of compounds I-3 and I-6 were 96.1 and 112.92 μM, respectively, which is more than 8 times their IC_50_ for alanine racemase. The results eliminated assay interference by the inhibitors.

### Cytotoxicity studies of three enzyme inhibitors with antimicrobial activity

To examine the cytotoxicity of these three enzyme inhibitors, we tested the inhibitors in HeLa cells. The TC_50_ values are shown in Table 1. The TC_50_ for the compounds I-3, I-6, and DCS were 157.7, 122.9 and 149.6 μg/ml [6], respectively. Compound I-2 exhibits strong cytotoxic effects and reduces the viability of HeLa cells up to 99% at 6.25 μg/ml. Compound I-2 is patulin, which had an IC_50_ value of 0.62 μg/ml against Caco-2 cells [25]. Compounds I-3 and I-6 are homogentisic acid and hydroquinone. The therapeutic indices (*T*_i_) of homogentisic acid, hydroquinone and DCS are 1.3, 1.54 and 5, respectively. The results indicate that homogentisic acid and hydroquinone are potential antimicrobial agents for *A. hydrophila.*

### Determination of inhibition constants

**Fig. 4:**
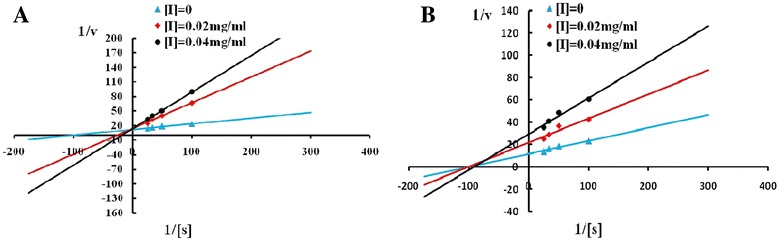
Lineweaver–Burk plots of Alr-2 activity assays at different concentrations of homogentisic acid (**a**) and hydroquinone (**b**)

To gain more insight into the inhibition of alanine racemase by homogentisic acid and hydroquinone, kinetic studies of inhibition were carried out. The Lineweaver–Burk plots were constructed as shown in Fig. 4. The *K*_m_ and *V*_max_ of Alr-2 without inhibitors were determined to be 1.1 mM and 0.94 mM/min, respectively. The Lineweaver–Burk profile of homogentisic acid was of a competitive type of inhibition, while the Lineweaver–Burk profile of hydroquinone was of a noncompetitive type of inhibition. The inhibition constants, *K*i, were determined to be 51.7 and 212 μM for homogentisic acid and hydroquinone, respectively. Based on the above data, we propose that homogentisic acid structurally resembles alanine but is not an alanine analog. Homogentisic acid competitively binds to the enzyme at the active site. Hydroquinone is not structurally similar to alanine and binds to the enzyme or enzyme-alanine at a site where alanine does not bind. Hydroquinone does not compete with alanine for the enzyme.

### Docking results of the inhibitors interacting with the Alr-2 protein

**Fig. 5:**
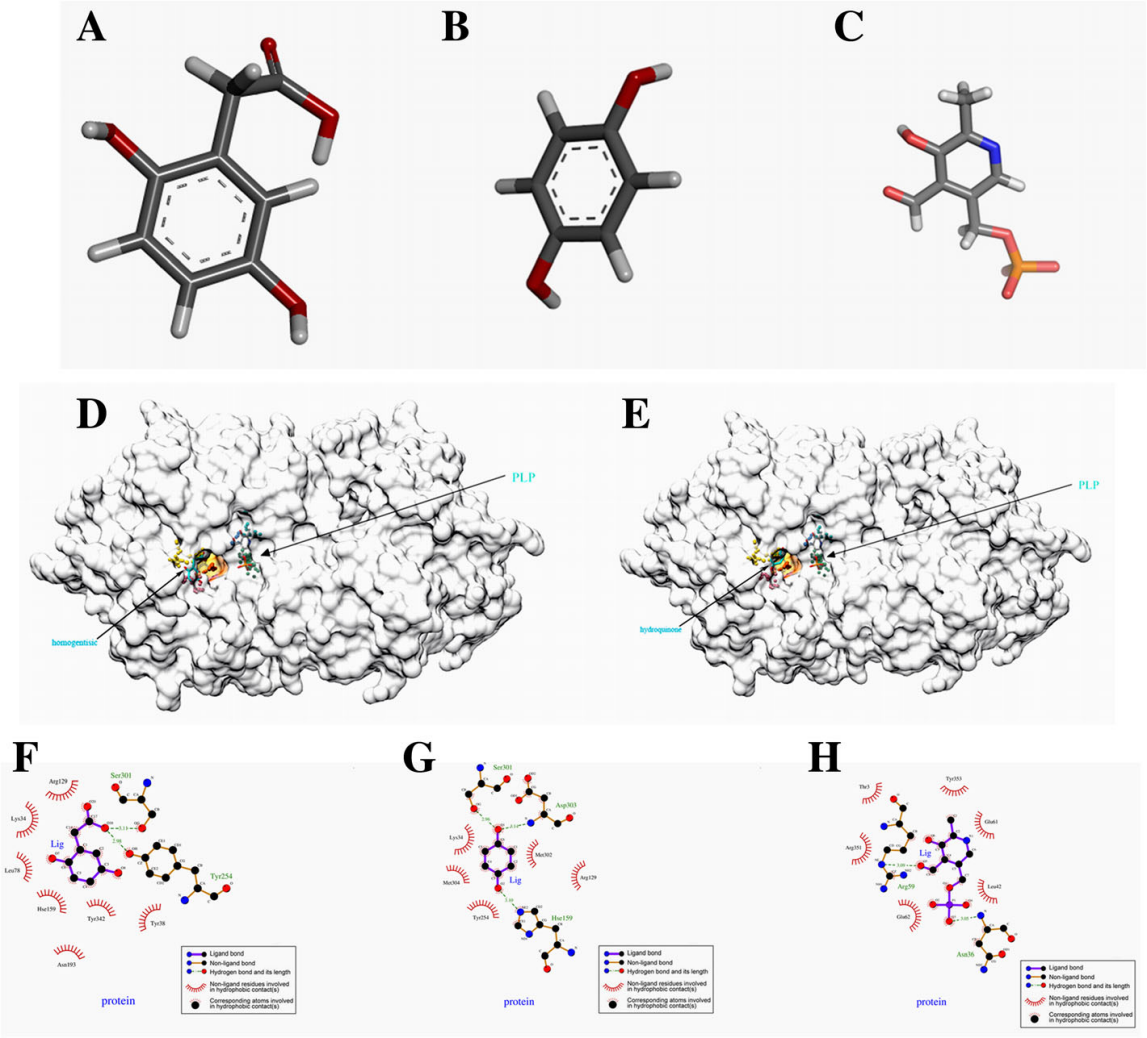
The molecular docking of Alr-2 with inhibitors. The 3D structure of homogentisic acid, hydroquinone and PLP was constructed using Corina online demonstration. The 3D structure of homogentisic acid (**a**), hydroquinone (**b**) and PLP (**c**) is shown. **d** Docking solution of homogentisic acid in the catalytic domain of Alr-2. **e** Docking solution of hydroquinone in the catalytic domain of Alr-2. The protein backbone of Alr-2 is shown with PLP interaction in the alpha model. **f** The 2D representation of homogentisic acid and its interaction with Alr-2 was analyzed using LIGPLOT. **g** The 2D representation of hydroquinone and its interaction with Alr-2 was analyzed using LIGPLOT. **h** The 2D representation of PLP and its interaction with Alr-2 was analyzed using LIGPLOT. NOTE: A H-bond is represented as a *dashed line*, and a spiked residue represents hydrophobic contacts

Structural coordinates for the Alr-2 protein were predicted using the Swiss-Model server. The best model, made with the protein structure (PDB IDs: 2rjh.1.A) as a template, had the highest structure quality. The GMQE (Global Model Quality Estimation) score was 0.81, and the QMEAN4 score was −2.49. AutoDock, which predicts the direct binding of proteins with small molecules, was used to verify the possible docking of the inhibitors to Alr-2. Docking results of inhibitors interacting with the Alr-2 protein were shown in Additional file 1: Figure S2-Figure S10. The data suggested that homogentisic acid almost occupies the catalytic active sites of Alr-2, while hydroquinone lie**s** near the active center of Alr-2 (Fig. 5d, e). A change in the binding residues will affect the interaction between the protein and molecules. When the inhibitors occupied the catalytic active sites of Alr-2, PLP could not bind covalently to the active sites. The binding sites of PLP are away from the catalytic center. The LIGPLOT analysis was then introduced to help us understand the in-depth interaction pattern between inhibitors, PLP and the active site residues of the Alr-2 protein. The data showed that homogentisic acid formed hydrogen bonds with Ser301 and Tyr254, which play a major role in the stabilization of the quinonoid intermediate and interacts with the catalytic Lys34 residue and active site residues Tyr38, Tyr342, Leu78, Arg129 and Hse159 in Alr-2. Hydroquinone formed hydrogen bonds with Ser301, Hse159, and Asp304, which are within the active sites of Alr-2, and interacts with the catalytic residues Lys34 and Tyr254 and residues Met304 and Arg129 in the active sites. PLP forms hydrogen bonds with Asn36 and Arg59 and interacts with the Leu42, Glu62, Glu62 Arg351 and Tyr353 residues, which are not in the active sites of the enzyme (Fig. 4f, g). This docking analysis gives a “theoretical quantitative” assessment of the binding efficiencies of inhibitors and proteins.

## Discussion

HTS has been used to screen for a new class of Alr inhibitors. Anthony et al. identified a novel series of Alr inhibitors, developed by high-throughput screening against *Mycobacterium tuberculosis* for non-substrate analogs, having different mechanisms of enzyme inhibition [6]. The novel enzyme inhibitor thiadiazolidinone was obtained, which inhibits the growth of methicillin-resistant *Staphylococcus aureus* (MRSA) [26], *M. tuberculosis* and *M. smegmatis* [27]. Willies SC et al. developed a high-throughput screening method for alanine racemase activity that can be identified in a solid format [28]. In this study, we improved the enzyme assay method of Willies to be a liquid phase assay for alanine racemase inhibitor screening and this assay has proved effective according to the screening and assay interference results.

The compound library is important for inhibitor screening. Anthony et al. used a library of 53,000 small molecules, including therapeutic compounds and natural compounds, for inhibitor screening in their study [6]. They found seven compounds that were active against *M. tuberculosis* and with minimal cytotoxicity against HeLa cells. In the present study, the compounds in the library were mainly the metabolites of microorganisms.

The majority of identified alanine racemase inhibitors, however, are suicide substrates that react with the enzyme cofactor and tend to inhibit other PLP-containing enzymes indiscriminately. In this study, we found several novel inhibitors that are not alanine analogs. The antibacterial activities and cytotoxicity noted for two of the inhibitors (homogentisic acid and hydroquinone) are non-substrate-like enzyme inhibitors and potential antibiotics for *A. hydrophila*. Homogentisic acid is a product of phenylalanine and tyrosine metabolism in a wide variety of higher organisms. Homogentisic acid actively inhibited the growth of eight species of gram-negative oral bacteria [29, 30]. Hydroquinone has antimicrobial activity against MRSA, methicillin-sensitive *S. aureus* (MSSA) and *E. coli* (BL) 21 in alkaline conditions [31, 32]. However, these compounds also have adverse side effects in humans. In patients with a rare autosomal recessive disorder, an accumulation of homogentisic acid leads to Alkaptonuria [33]. Hydroquinone is a toxic compound of *Agaricus hondensishas* and widely used in skin lightening (bleaching) cosmetics and toiletries. Abuse of hydroquinone will result in adverse side effects and complications [34, 35]. It is the first time the antimicrobial activity of homogentisic acid and hydroquinone against *A. hydrophila* has been confirmed.

The earliest drug development for alanine racemase was carried out in the absence of a crystal structure and resulted in the development of a small, covalent inhibitor, cycloserine. Cycloserine interacts with the enzyme-bound PLP and interferes with the catalytic process of the enzyme [36]. Although the MIC values of homogentisic acid and hydroquinone is 3-5 times higher than that of DCS, they directly interact with the active sites of alanine racemase. These compounds, unlike DCS, do not inactivate other unrelated PLP-dependent enzymes and are potential antimicrobials against *A. hydrophila*.

The small-molecule screening approach has been successfully used for HTS of bacterial targets. Many effective inhibitors to these targets were identified and these compounds inhibit the target by many mechanisms [7]. Alanine racemase is an important target for antibacterial drug design as it is a key enzyme in peptidoglycan biosynthesis and bacterial cell wall formation. The reason most inhibitors of alanine racemase are not used clinically is because they lack specificity and target other PLP-dependent enzymes. One of the representative inhibitors is DCS, a restricted drug against tuberculosis with serious side effects on the nervous system [37]. This emphasizes the need for the development of new inhibitors for Alr with greater specificity that may in turn translate into less toxicity to humans.

Numerous molecular modeling studies of alanine racemase inhibitors have been published. Scaletti ER et al. successfully purified, crystallized, determined the structure and performed a kinetic characterization of *Staphylococcus aureus* alanine racemase from the Mu50 strain (AlrSas), which exhibits resistance to both methicillin and glycopeptide antibiotics. Structural examination indicates that the active site binding pocket, dimer interface and active site entryway of the enzyme are potential targets for structure-aided inhibitor design. This structural and biochemical information provides a template for future structure-based drug-development efforts targeting AlrSas [38]. To determine the specific inhibitors that act on the active sites of the enzyme from *A. hydrophila*, we performed a systematic computational analysis to identify inhibitor-protein interactions through molecular docking. Finally, we identified two compounds that could interact with the active sites of alanine racemase and would make effective inhibitors. These inhibitors have never been reported.

## Conclusions

The results presented in this study suggested an efficient combination of experimental and computational methods for the screening of *Aeromonas hydrophila* alanine racemase Alr-2 inhibitors. The inhibitor-protein interactions exhibited by the inhibitors could be applied to the development of new antibiotics against *A. hydrophila*.

## Additional file

Additional file 1:
**Figure S1.** The second screening dataset of ten compounds screened in the inhibitor screening. **Figure S2.** The RMSD fluctuation profile of the modeled protein over 20 ns MD simulations. **Figure S3.** The superposed structures clustered from MD simulations. **Figure S4.** The top binding mode of hydroquinone calculated from Dock6. **Figure S5.** The top binding mode of hydroquinone calculated from AutoDock Vina. **Figure S6.** The top binding mode of hydroquinone calculated from AutoDock 4. **Figure S7.** The top binding mode of homogentisic acid calculated from Dock6. **Figure S8.** The top binding mode of the homogentisic acid calculated from AutoDock Vina. **Figure S9.** The top binding mode of the homogentisic acid calculated from AutoDock 4. Docking results of the Alr-2 protein interacting with alanine. **Figure S10.** The top binding mode of the alanine calculated from AutoDock Vina. (DOC 4146 kb)

## Abbreviations

*A. hydrophila*: *Aeromonas hydrophila*
AAP: 4-aminoantipyrine
AMP: Ampicillin
CFU: Colony-forming units
DAAO: d-amino acid oxidase
DCS: d-cycloserine
DMSO: Dimethyl sulfoxide
GMQE: Global model quality estimation
homogentisic acid: 2,5-Dihydroxyphenylacetic acid
HRP: Horseradish peroxidase
IC_50_: 50% Inhibitory concentration
*K*_i_: Inhibition constant
LB: Luria-Bertani
MICs: Minimal inhibitory concentrations
MRSA: Methicillin-resistant *Staphylococcus aureus*
MSSA: Methicillin sensitive *S. aureus*
MTT: 3-(4,5-dimethyl-2-thiazole)-2,5-diphenyl-2H-tetrazolium bromide
PLP: Pyridoxal-5′-phosphate
S/B: Signal-to-background ratio
S/N: Signal-to-noise ratio
*T*_i_: Therapeutic indices
TOOS: *N*-ethyl-*N*-(2-hydroxy-3-sulfopropyl)-*m*-toluidine sodium salt

## References

[1] Singh V, Chaudhary DK, Mani I, Jain R, Mishra BN (2013). Development of diagnostic and vaccine markers through cloning, expression, and regulation of putative virulence-protein-encoding genes of *Aeromonas hydrophila*. J Microbiol.

[2] Hossain MJ, Waldbieser GC, Sun D, Capps NK, Hemstreet WB, Carlisle K (2013). Implication of lateral genetic transfer in the emergence of *Aeromonas hydrophila* isolates of epidemic outbreaks in channel catfish. PLoS One.

[3] Azam MA, Jayaram U (2015). Inhibitors of alanine racemase enzyme: a review. J Enzyme Inhib Med Chem.

[4] Sharma V, Gupta P, Dixit A (2008). In silico identification of putative drug targets fromdifferent metabolic pathways of *Aeromonas hydrophila*. In Silico Biol.

[5] di Salvo ML, Florio R, Paiardini A, Vivoli M, D'Aguanno S, Contestabile R (2013). Alanine racemase from *Tolypocladium inflatum*: a key PLP-dependent enzyme in cyclosporine biosynthesis and a model of catalytic promiscuity. Arch Biochem Biophys.

[6] Anthony KG, Strych U, Yeung KR, Shoen CS, Perez O, Krause KL, Cynamon MH, Aristoff PA, Koski RA (2011). New classes of alanine racemase inhibitors identified by high-throughput screening show antimicrobial activity against *Mycobacterium tuberculosis*. PLoS One.

[7] Im H, Sharpe ML, Strych U, Davlieva M, Krause KL (2011). The crystal structure of alanine racemase from *Streptococcus pneumoniae*, a target for structure-based drug design. BMC Microbiol.

[8] Kim MG, Strych U, Krause K, Benedik M, Kohn H (2003). Evaluation of amino-substituted heterocyclic derivatives as Alanine Racemase inhibitors. Med Chem Res.

[9] Kim MG, Strych U, Krause K, Benedik M, Kohn H (2003). N (2)-substituted D, L-cycloserine derivatives: synthesis and evaluation as alanine racemase inhibitors. J Antibiot.

[10] Toney MD (2005). Reaction specificity in pyridoxal phosphate enzymes. Arch Biochem Biophys.

[11] Radkov AD, Moe LA (2014). Bacterial synthesis of D-amino acids. Appl Microbiol Biotechnol.

[12] Li N, Sun YM, Tian LY, Zhao BH (2009). The isolation and identification of *Aeromonas hydrophila*. J Hebei Normal Univ /Nat Sci Ed.

[13] Liu D, Zhang L, Xue W, Wang YP, Ju JS, Zhao BH. Knockout of the alanine racemase gene in *Aeromonas hydrophila* HBNUAh01 results in cell wall damage and enhanced membrane permeability. FEMS Microbiol Lett. 2015;362: fnv089. doi: 10.1093/femsle/fnv089. 10.1093/femsle/fnv089 26040590

[14] Liu D, Liu XP, Zhang L, Jiao HW, Ju JS, Zhao BH (2015). Biochemical characteristics of an alanine racemase from *Aeromonas hydrophila* HBNUAh01. Microbiology.

[15] Zhang JH, Chung TD, Oldenburg KR (1999). A simple statistical parameter for use in evaluation and validation of high throughput screening assays. J Biomol Screen.

[16] Clinical Laboratory Standards Institute. Antimicrobial disk and dilution susceptibility tests for bacteria isolated from animals. Approved standard, 3rd ed. Wayne: CLSI; 2008. CLSI Document M31-A3.

[17] Dal Pozzo M, Viégas J, Santurio DF, Rossatto L, Soares IH, Alves SH, Costa MM (2011). Atividade antimicrobiana de óleos essenciais decondimentos frente a *Staphylococcus* sp. isolados de mastite caprina. Ciênc Rural.

[18] Feng ZY, Barletta RG (2003). Roles of *Mycobacterium smegmatis* D-alanine:D-alanine ligase and D-alanine racemase in the mechanisms of action of and resistance to the peptidoglycan inhibitor D-cycloserine. Antimicrob Agents Chemother.

[19] Mosmann T (1983). Rapid colorimetric assay for cellular growth and survival: application to proliferation and cytotoxicity assays. J Immunol Methods.

[20] Goodsell DS, Olson AJ (1990). Automated docking of substrates to proteins by simulated annealing. Proteins.

[21] Wu D, Hu T, Zhang L, Chen J, Du J, Ding J, Jiang H, Shen X (2008). Residues Asp164 and Glu165 at the substrate entryway function potently in substrate orientation of alanine racemase from *E. coli*: enzymatic characterization with crystal structure analysis. Protein Sci.

[22] Arnold K, Bordoli L, Kopp J, Schwede T (2006). The SWISS-MODEL workspace: a web-based environment for protein structure homology modelling. Bioinformatics.

[23] Ni Z, Jin X, Zhou P, Wu Q, Lin XF (2011). A combination of computational and experimental approaches to investigate the binding behavior of *B.sub* lipase a mutants with substrate *p*NPP. Mol Inform.

[24] Trott O, Olson AJ (2010). AutoDock Vina: improving the speed and accuracy of docking with a new scoring function, efficient optimization, and multithreading. J Comput Chem.

[25] Ciustea M, Mootien S, Rosato AE, Perez O, Cirillo P, Yeung KR, Ledizet M, Cynamon MH, Aristoff PA, Koski RA, Kaplan PA, Anthony KG (2012). Thiadiazolidinones: a new class of alanine racemase inhibitors with antimicrobial activity against methicillin-resistant *Staphylococcus aureus*. Biochem Pharmacol.

[26] Willies SC, White JL, Turner NJ (2012). Development of a high-throughput screening method for racemase activity and its application to the identification of alanine racemase variants with activity towards l-arginine. Tetrahedron.

[27] Lupescu A, Jilani K, Zbidah M, Lang F (2013). Patulin-induced suicidal erythrocyte death. Cell Physiol Biochem.

[28] Lee Y, Mootien S, Shoen C, Destefano M, Cirillo P, Asojo OA, Yeung KR, Ledizet M, Cynamon MH, Aristoff PA, Koski RA, Kaplan PA, Anthony KG (2013). Inhibition of mycobacterial alanine racemase activity and growth by thiadiazolidinones. Biochem Pharmacol.

[29] Frases S, Salazar A, Dadachova E, Casadevall A (2007). *Cryptococcus neoformans* Can utilize the bacterial melanin precursor homogentisic acid for fungal Melanogenesis. Appl Environ Microbiol.

[30] Fukamachi H, Matsumoto C, Omiya Y, Arimoto T, Morisaki H, Kataoka H, Kadena M, Funatsu T, Fukutake M, Kase Y, Kuwata H (2015). Effects of Hangeshashinto on growth of oral microorganisms. Evid Based Complement Alternat Med.

[31] Chaaban I, Khawass ESME, Mahran MA, Razik HAAE, Salamouni NSE, Wahab AEA (2013). Synthesis and biological evaluation of novel hydroquinone dimethyl ethers as potential anticancer and antimicrobial agents. Med Chem Res.

[32] Guo JY, Hong ZG, Zhang LL, Wu HG, Dou CZ (2014). Preliminary study the effects of semiquinone radicals on bacteriostatic activity. China J Tradit Chin Med Pharm.

[33] Phornphutkul C, Introne WJ, Perry MB (2002). Natural history of alkaptonuria. N Engl J Med.

[34] Olumide YM, Akinkugbe AO, Altraide D, Mohammed T, Ahamefule N, Ayanlowo S, Onyekonwu C, Essen N (2008). Complications of chronic use of skin lightening cosmetics. Int J Dermatol.

[35] Joval E, Kroeger P, Towers N (1996). Hydroquinone: the toxic compound of *Agaricus hondensis*. Planta Med.

[36] Weinstein L. Antimicrobial agents: drugs used in the chemotherapy of tuberculosis and leprosy. In The pharmacological basis of therapeutics. 5 edition. In: Goodman LS, Gilman A, Editors. New York: Macmillan Publishing Co. Inc; 1975:1201-1223.

[37] Newton RW. Side effects of drugs used to treat tuberculosis. Scott Med J. 1975;20:47–9. 10.1177/003693307502000204 47648

[38] Scaletti ER, Luckner SR, Krause KL. Structural features and kinetic characterization of alanine racemase from *Staphylococcus aureus *(Mu50). Acta Crystallogr D Biol Crystallogr. 2012;68:82-92. 10.1107/S0907444911050682 PMC3245724 22194336

