# Supplementary material for: Selection and characterization of alanine racemase inhibitors against Aeromonas hydrophila
Source: BMC Microbiol. 2017 May 25;17:122. doi: 10.1186/s12866-017-1010-x (PMC5445283; doi:10.1186/s12866-017-1010-x)
Supplement: Additional file 1: — Figure S1. The second screening dataset of ten compounds screened in the inhibitor screening. Figure S2. The RMSD fluctuation profile of the modeled protein over 20 ns MD simulations. Figure S3. The superposed structures clustered from MD simulations. Figure S4. The top binding mode of hydroquinone calculated from Dock6. Figure S5. The top binding mode of hydroquinone calculated from AutoDock Vina. Figure S6. The top binding mode of hydroquinone calculated from AutoDock 4. Figure S7. The top binding mode of homogentisic acid calculated from Dock6. Figure S8. The top binding mode of the homogentisic acid calculated from AutoDock Vina. Figure S9. The top binding mode of the homogentisic acid calculated from AutoDock 4. Docking results of the Alr-2 protein interacting with alanine. Figure S10. The top binding mode of the alanine calculated from AutoDock Vina. (DOC 4146 kb) [file 12866_2017_1010_MOESM1_ESM.doc]

**Additional file 1**

**1. The second screening dataset of ten compounds screened in the inhibitor screening.**


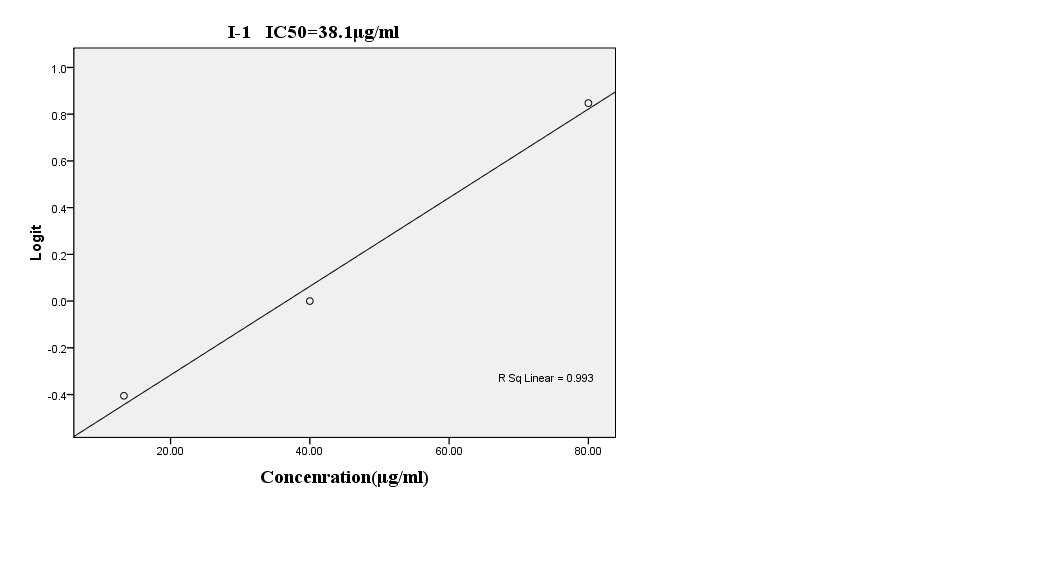

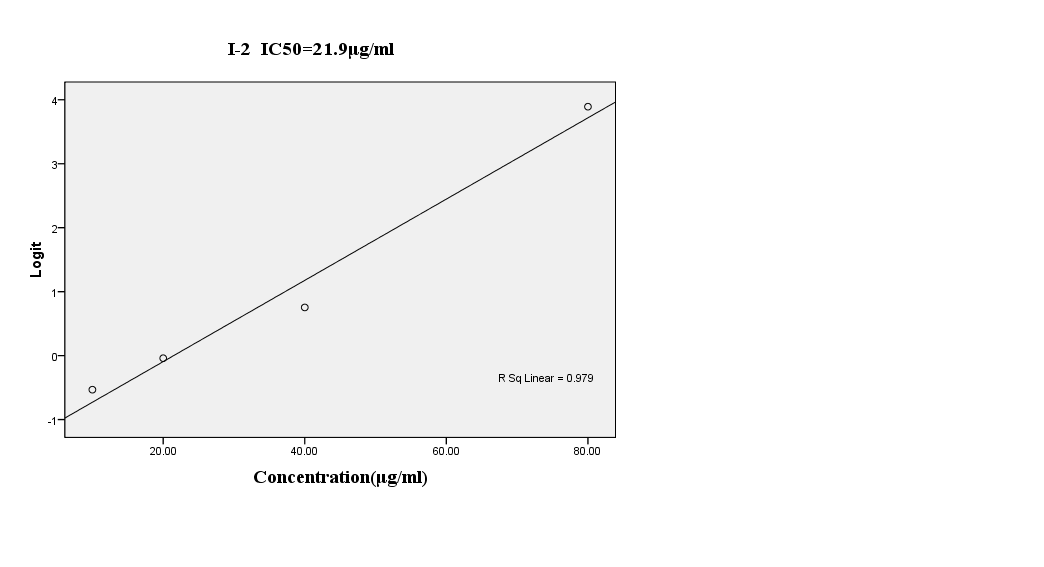

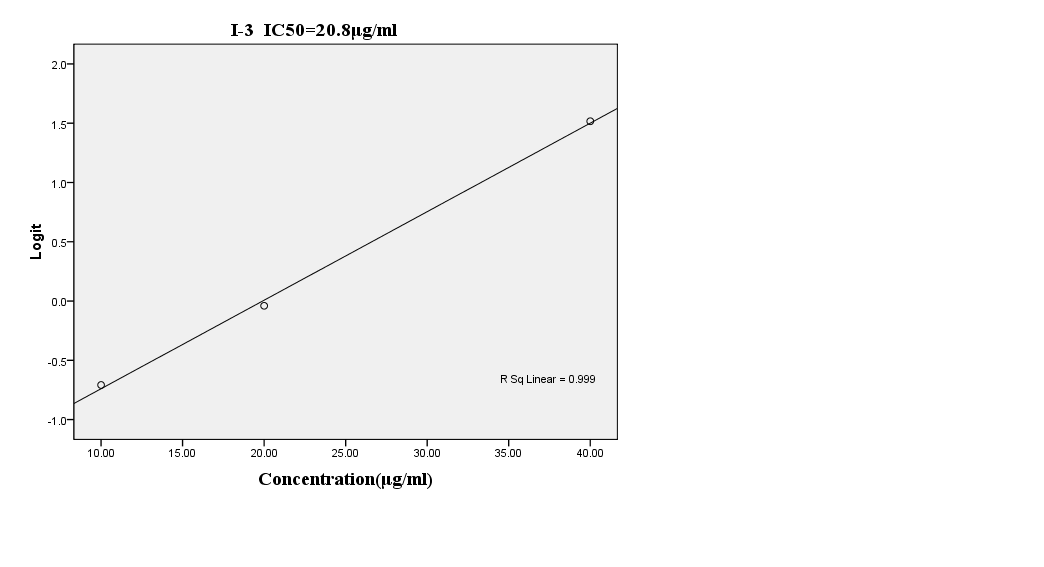

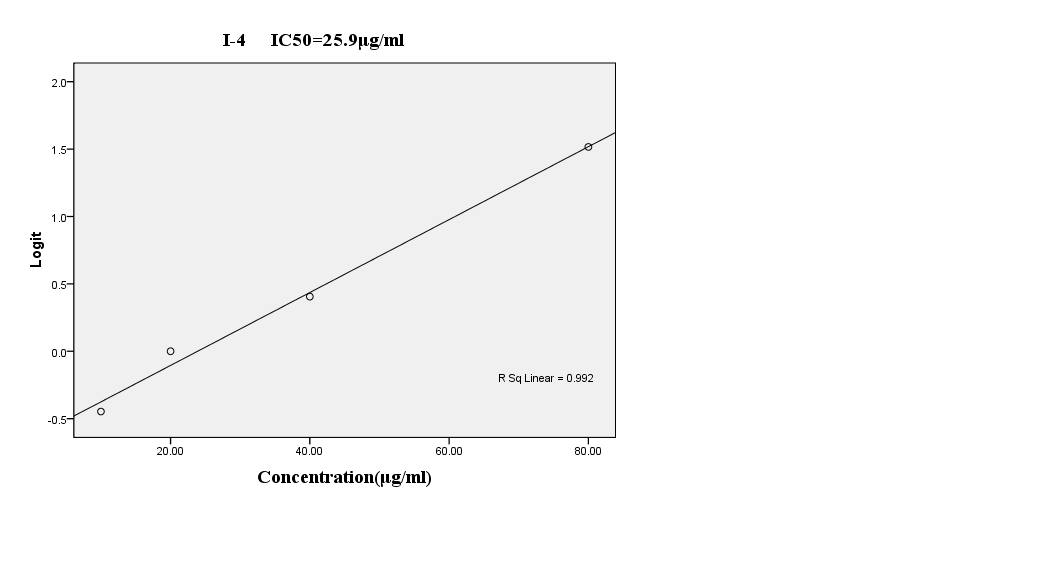

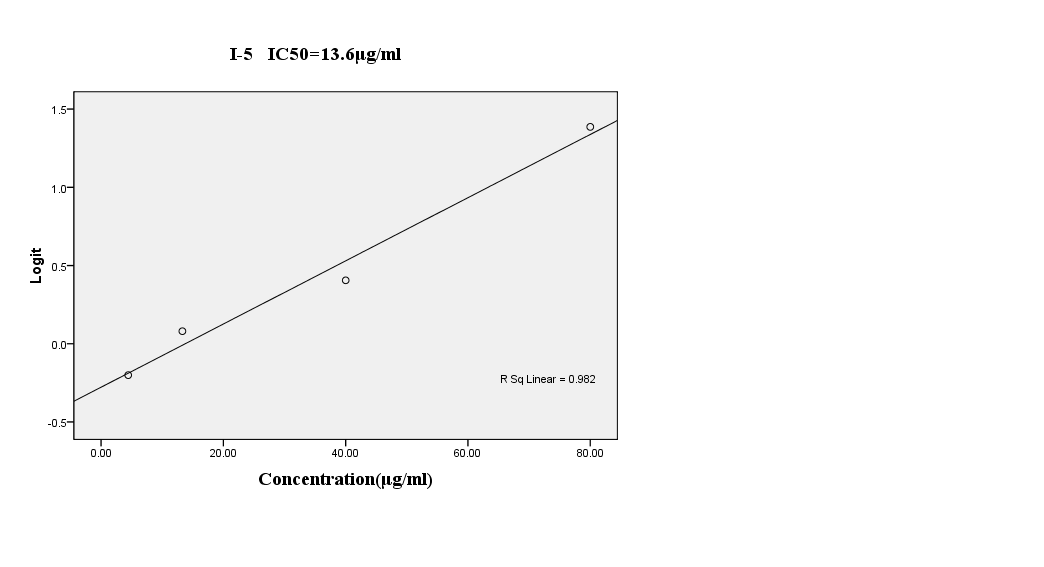

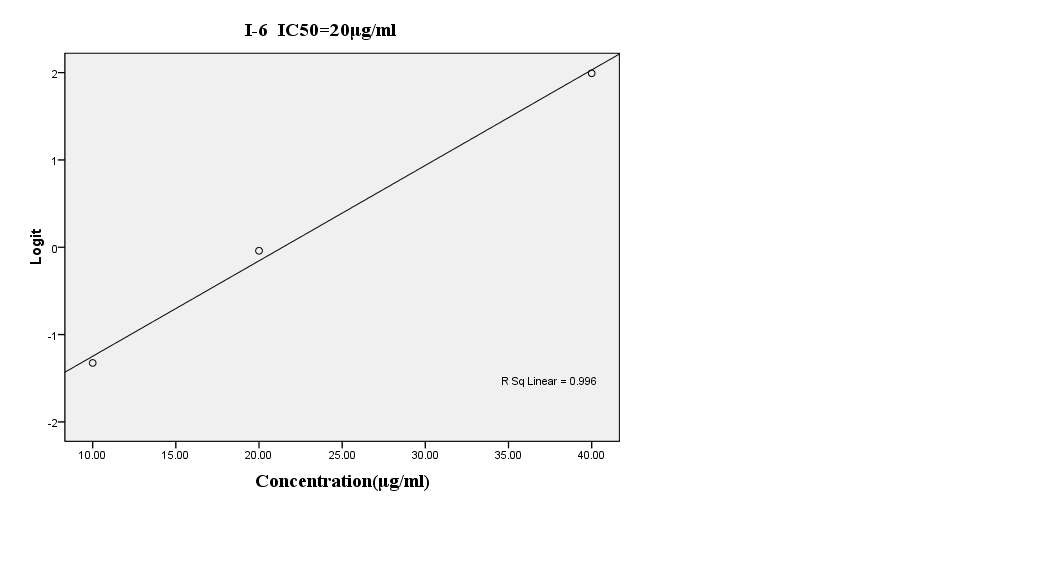

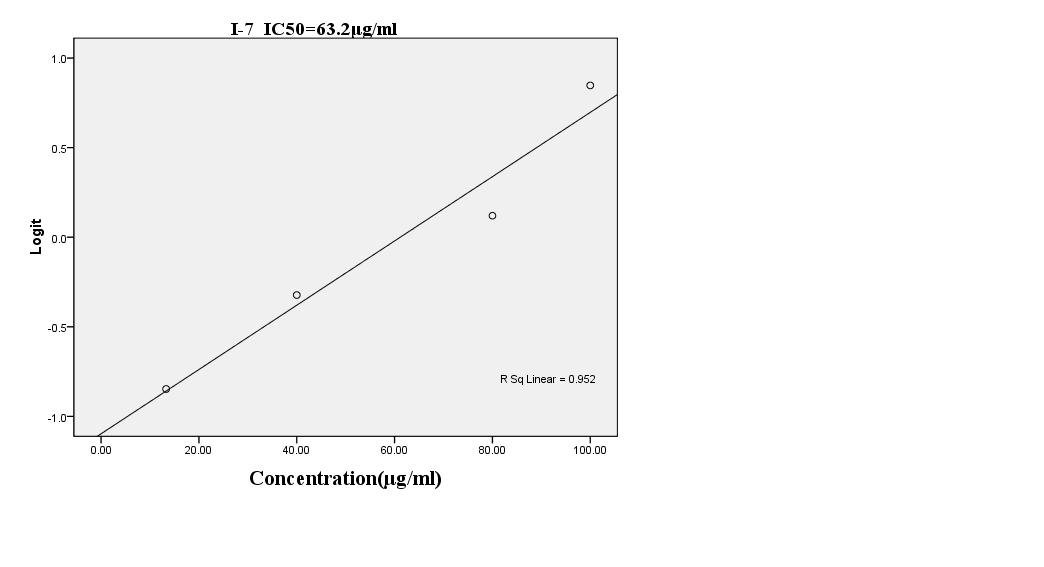

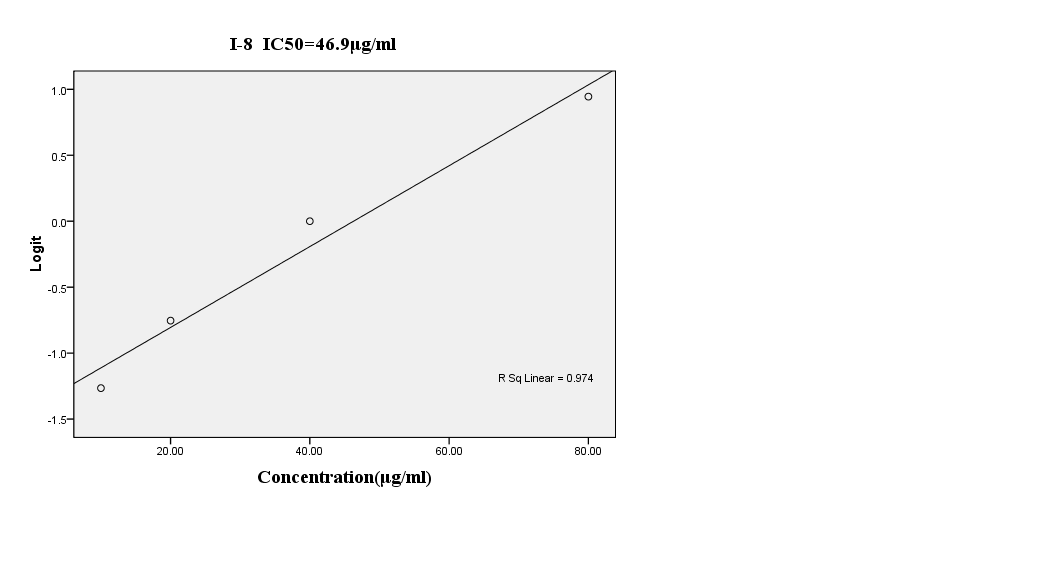

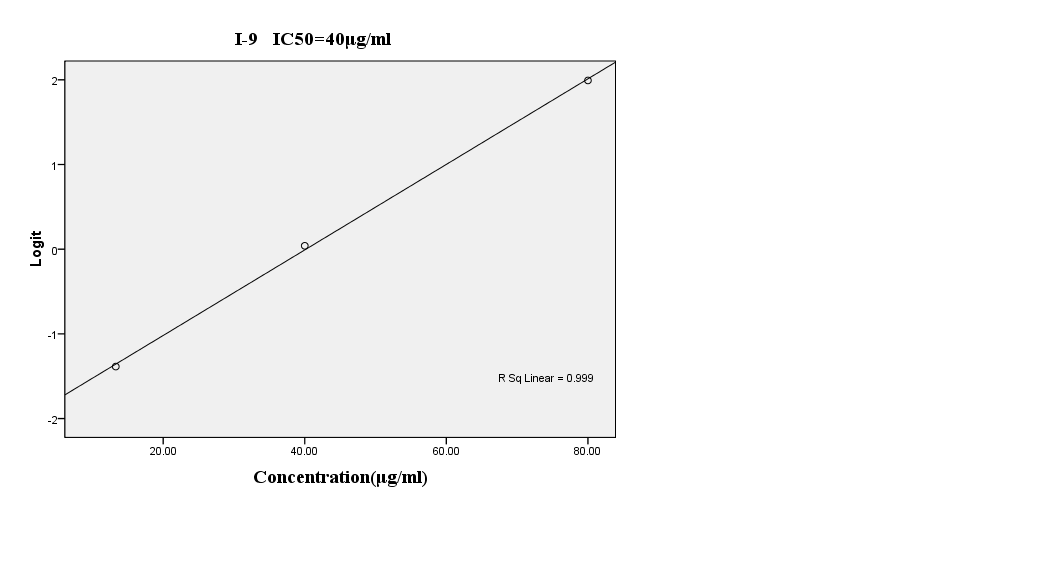

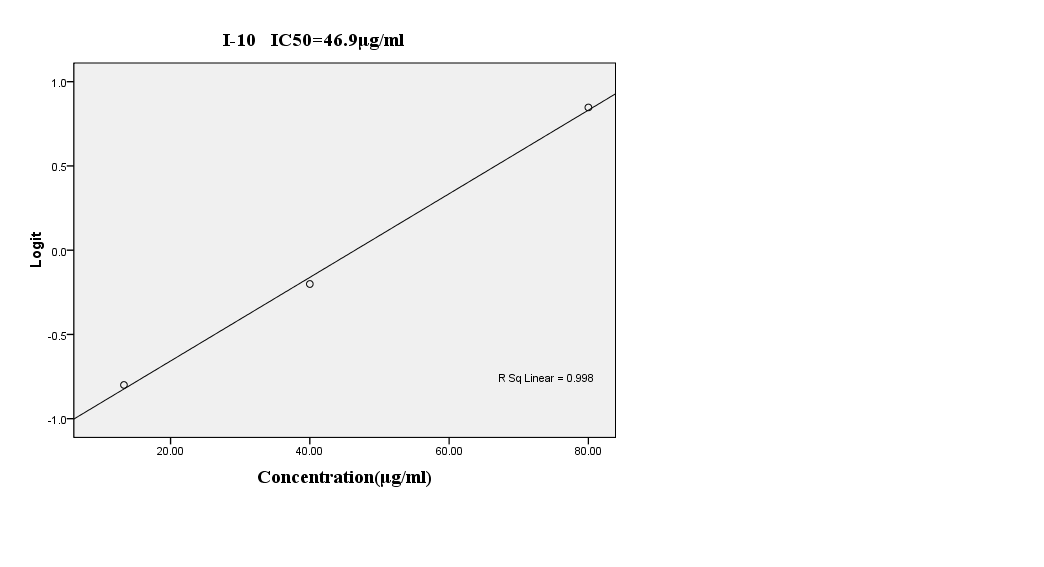

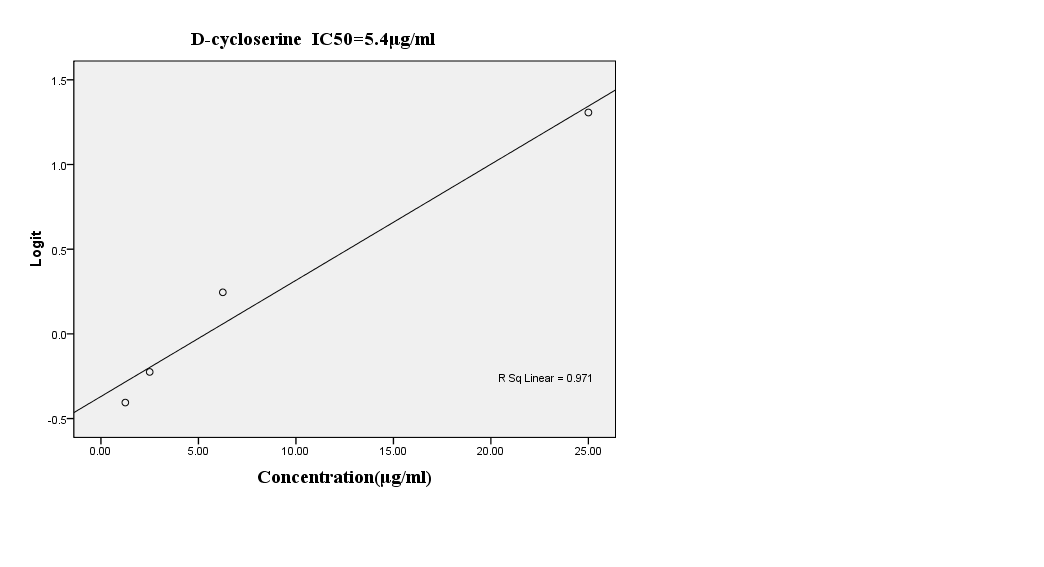


Fig. S1 The second screening dataset of ten compounds screened in the inhibitor screening.

**2. Docking results of inhibitors interacting with the Alr-2 protein**

For our homology modeling of alanine racemase, using the Swiss-Model[1], we performed 20 ns MD simulations (using Amber16 software) of this model to observe the protein dynamics and test the stability of our model. As shown in Fig. S2, it is suggested from the RMSD profile that the system was found to be relatively stable over the simulation period.


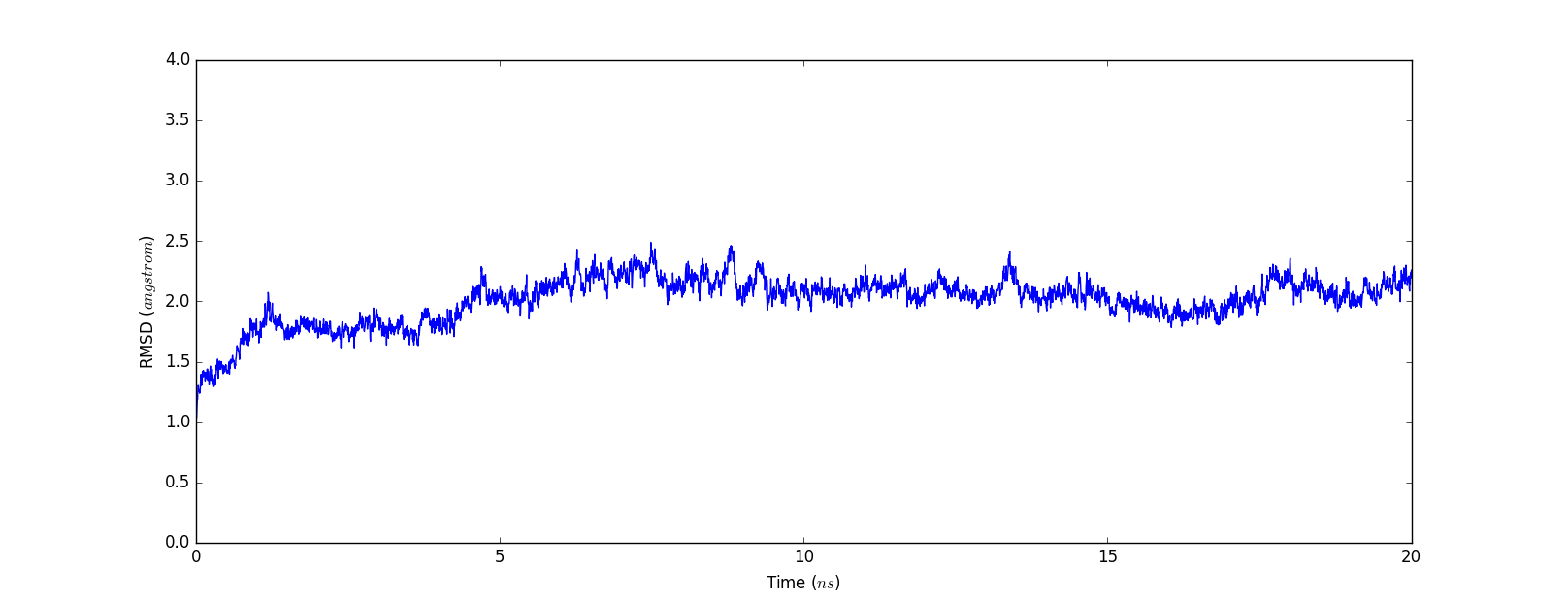


Fig. S2 The RMSD fluctuation profile of the modeled protein over 20 ns MD simulations


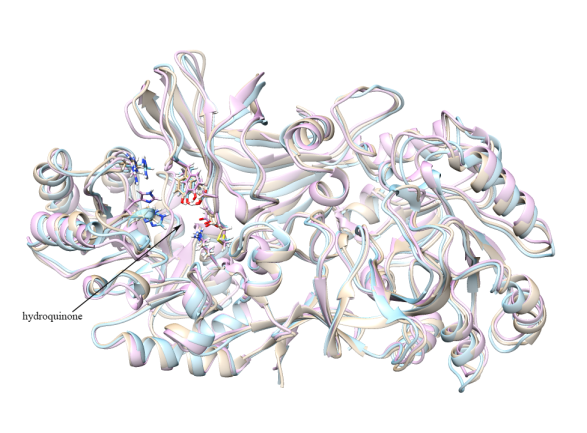

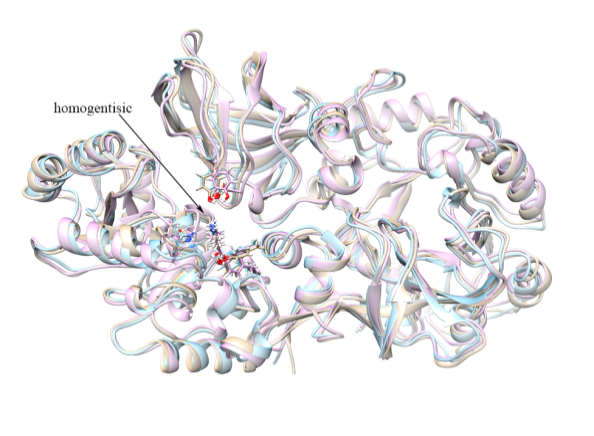


Fig. S3 The superposed structures clustered from MD simulations.

Then, we analyzed the variation of the binding pocket regions using a clustered structure calculated from the MD simulation. As shown in Fig. S3, the top 3 (identified to occupy with 85% probability the previous 10 ns MD structures) clustered structures were superposed with little fluctuation due to thermodynamics. The interacting residues at the binding pocket region show no obvious conformational changes, indicating that our homology model is well constructed and can be used to perform docking.

The purpose of the in silico docking performed in our experiment is to find the possible binding site of alanine racemase to accommodate our inhibitors. Here, we additionally performed docking by using Dock6 [2] and AutoDock 4 software [3]. Both are use a flexible docking algorithm. The results were compared with our previous docking results using AutoDock Vina [4]. All the available docking software that we have used are freely available and friendly to academic users.


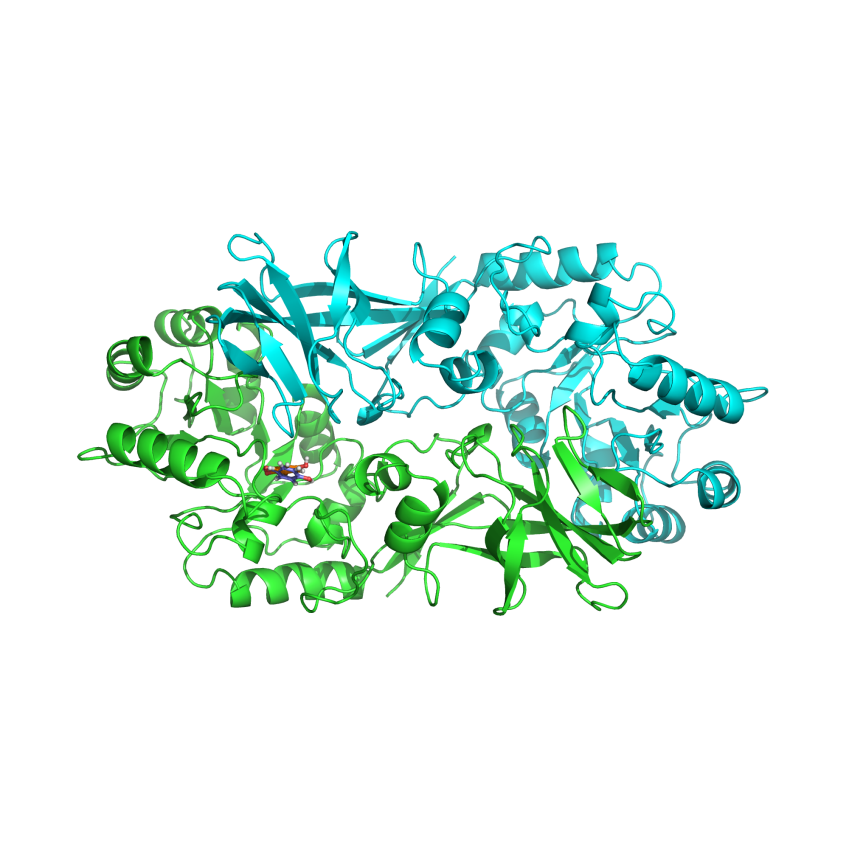

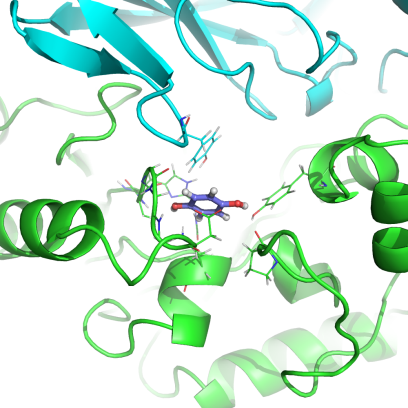


Fig. S4 The top binding mode of hydroquinone calculated from Dock6.


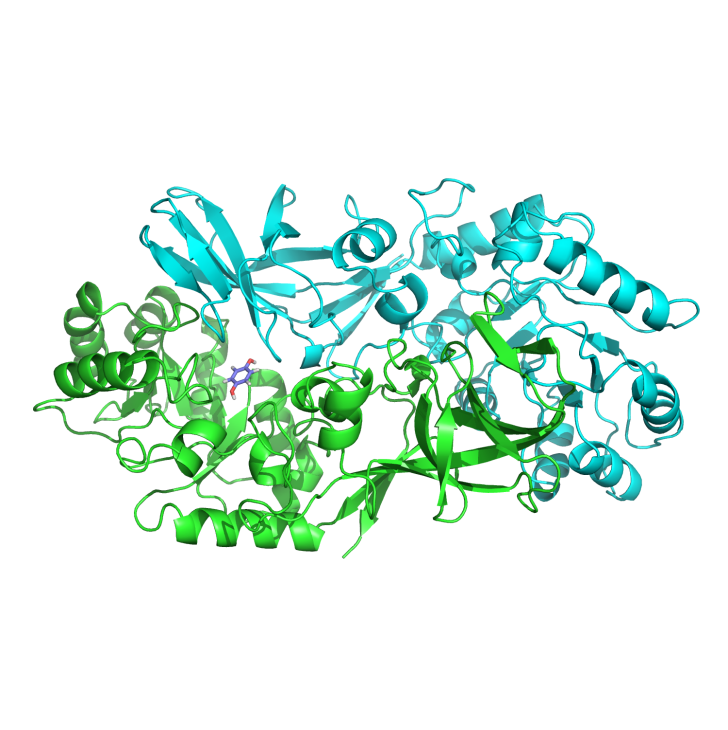

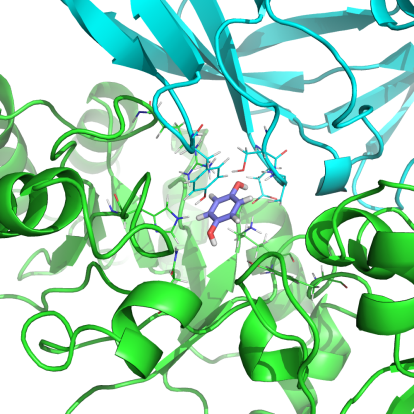


Fig. S5 The top binding mode of hydroquinone calculated from AutoDock Vina.


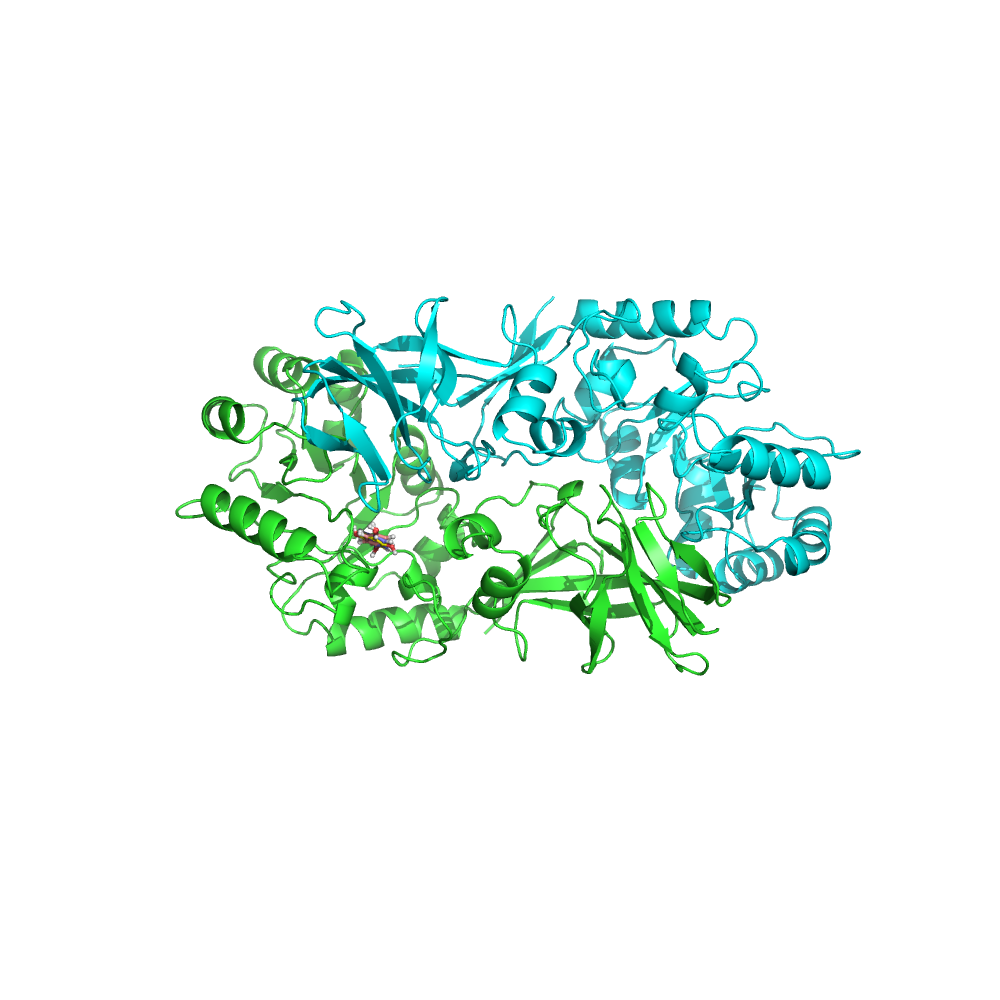

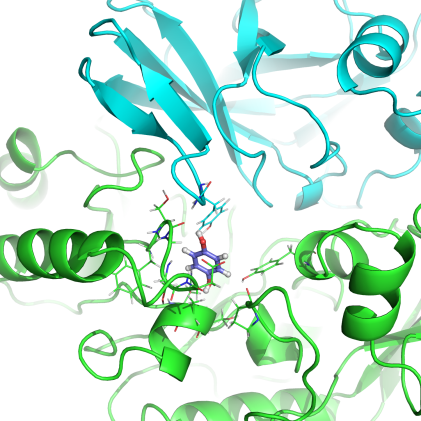


Fig. S6 The top binding mode of hydroquinone calculated from AutoDock 4.


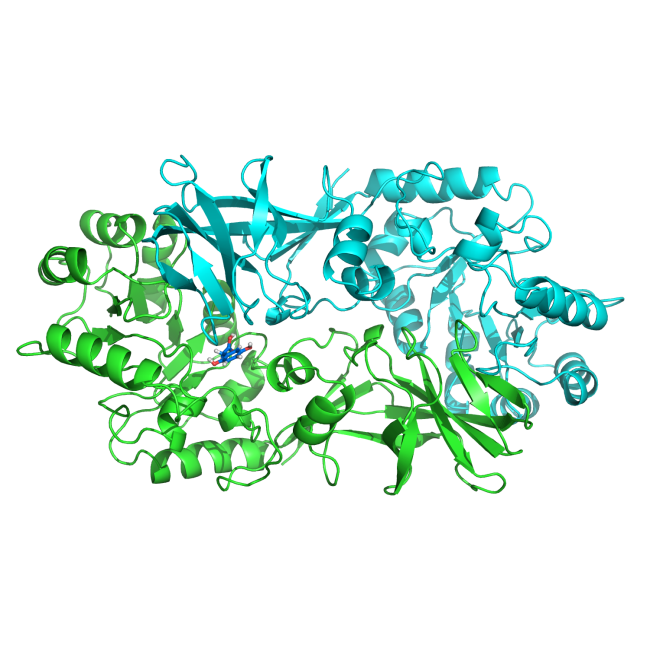

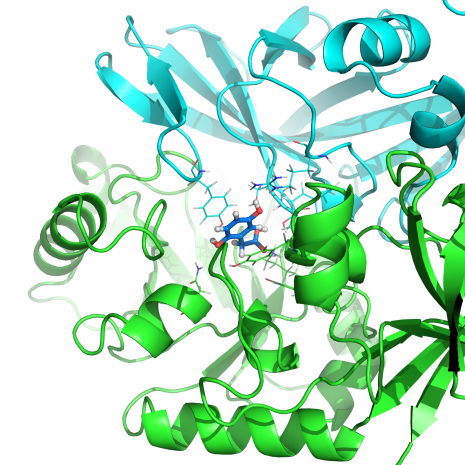


Fig. S7 The top binding mode of homogentisic acid calculated from Dock6


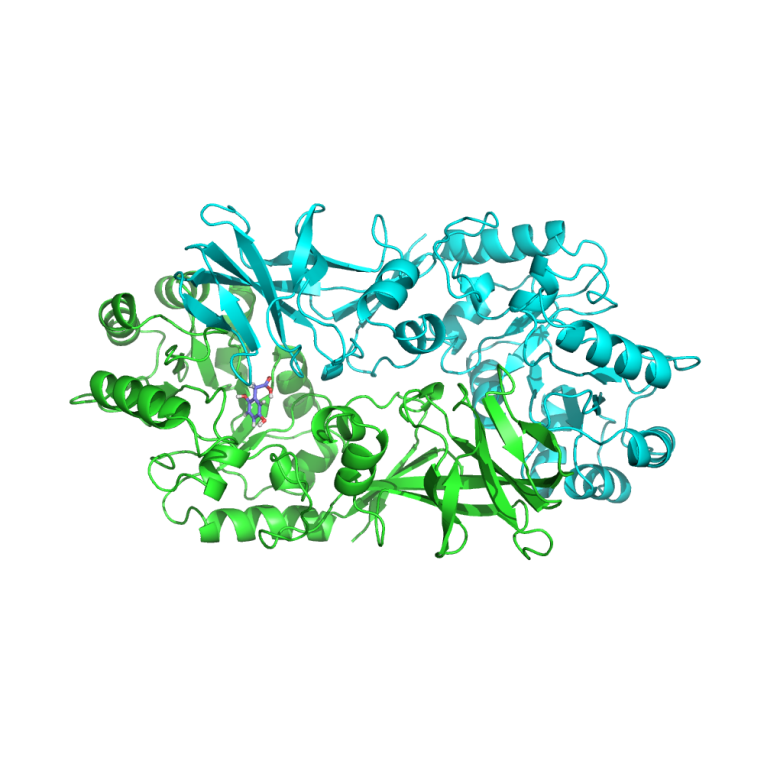

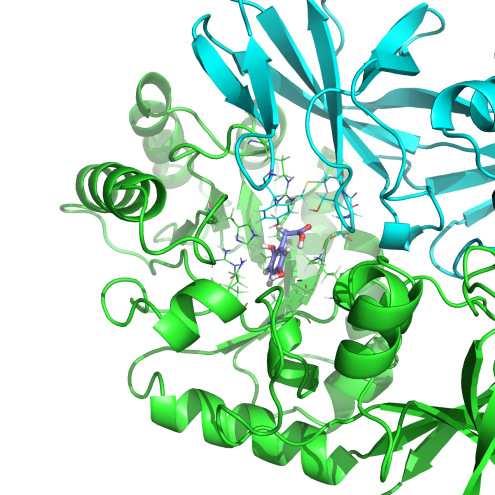


Fig. S8 The top binding mode of the homogentisic acid calculated from AutoDock Vina


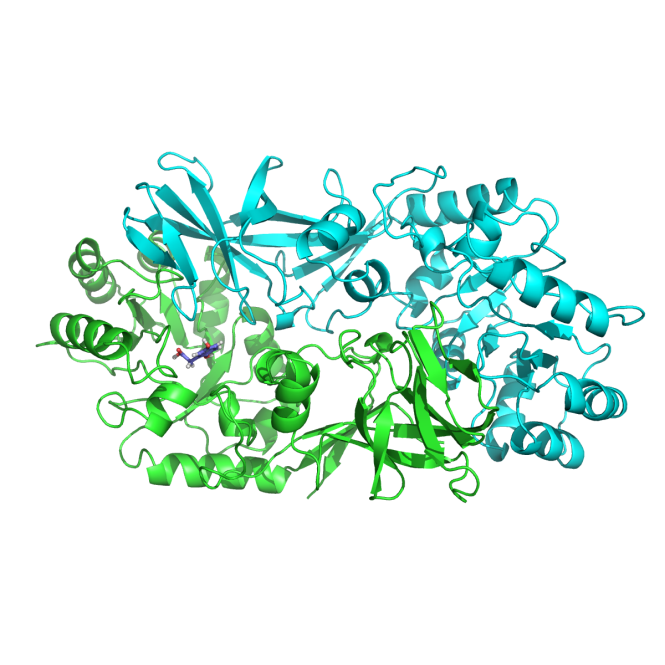

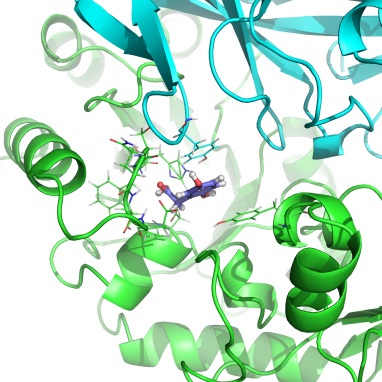


Fig. S9 The top binding mode of the homogentisic acid calculated from AutoDock 4

As shown in Fig. S4, S5 and S6, the hydroquinone molecule binds to the same binding pocket of alanine racemase. Common Pi-Pi interactions and hydrogen bonding are involved in the interacting residues with hydroquinone, although the conformation of hydroquinone varies between the three docking results. Similar results were observed for homogentisic acid binding modes (Fig. S7, S8 and S9). The binding sites of the two ligands are close to each other, and compared with the experiment results, this suggest that one ligand is located at the active site, while the other is near the active site and can cause a conformational change of the active site to indirectly inhibit alanine racemase. Further optimization can be done by using MD simulations. Here, our experiment results with computational in-silico docking methods show that alanine racemase can be a target of interest for further exploration.

**3. Docking results of the Alr-2 protein interacting with alanine**

We offer the docking result of the substrate location in fig. S10. As shown by the top binding mode of the homogentisic acid and hydroquinone, the binding sites of homogentisic acid and hydroquinone are also located around the catalytic sites binding to the alanine. Thus, we can see the steric hindrance for these inhibitors in fig. S10.


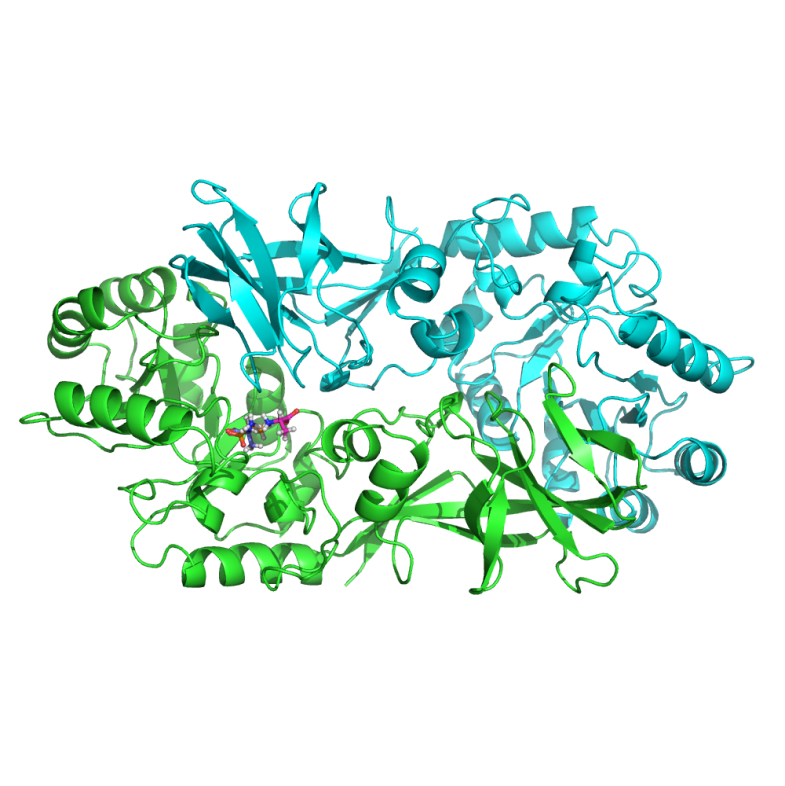

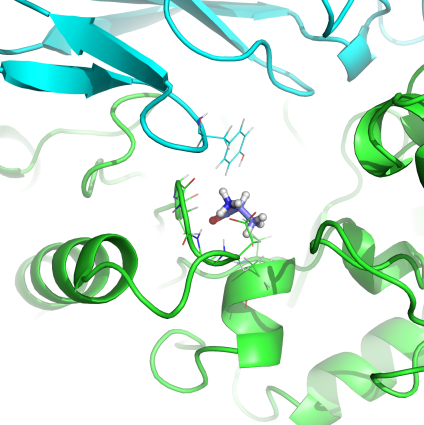


Fig. S10 The top binding mode of the alanine calculated from AutoDock Vina
